# Homodyne Solid-State Biased Coherent Detection of Ultra-Broadband Terahertz Pulses with Static Electric Fields

**DOI:** 10.3390/nano11020283

**Published:** 2021-01-22

**Authors:** Alessandro Tomasino, Riccardo Piccoli, Yoann Jestin, Boris Le Drogoff, Mohamed Chaker, Aycan Yurtsever, Alessandro Busacca, Luca Razzari, Roberto Morandotti

**Affiliations:** 1 INRS-EMT, 1650 Boulevard Lionel-Boulet, Varennes, QC J3X 1S2, Canada; riccardo.piccoli@emt.inrs.ca (R.P.); yoann.jestin@emt.inrs.ca (Y.J.); ledrogof@emt.inrs.ca (B.L.D.); chaker@emt.inrs.ca (M.C.); Aycan.Yurtsever@emt.inrs.ca (A.Y.); razzari@emt.inrs.ca (L.R.); 2 DEIM, University of Palermo, Edificio 9, Viale delle Science, 90128 Palermo, Italy; alessandro.busacca@unipa.it; 3 Institute of Fundamental and Frontier Sciences, University of Electronic Science and Technology of China, Chengdu 610054, China

**Keywords:** THz pulse detection, solid-state device, four-wave mixing

## Abstract

We present an innovative implementation of the solid-state-biased coherent detection (SSBCD) technique, which we have recently introduced for the reconstruction of both amplitude and phase of ultra-broadband terahertz pulses. In our previous works, the SSBCD method has been operated via a heterodyne scheme, which involves demanding square-wave voltage amplifiers, phase-locked to the THz pulse train, as well as an electronic circuit for the demodulation of the readout signal. Here, we demonstrate that the SSBCD technique can be operated via a very simple homodyne scheme, exploiting plain static bias voltages. We show that the homodyne SSBCD signal turns into a bipolar transient when the static field overcomes the THz field strength, without the requirement of an additional demodulating circuit. Moreover, we introduce a differential configuration, which extends the applicability of the homodyne scheme to higher THz field strengths, also leading a two-fold improvement of the dynamic range compared to the heterodyne counterpart. Finally, we demonstrate that, by reversing the sign of the static voltage, it is possible to directly retrieve the absolute THz pulse polarity. The homodyne configuration makes the SSBCD technique of much easier access, leading to a vast range of field-resolved applications.

## 1. Introduction

Coherent detection of ultra-broadband terahertz (THz) pulses (electromagnetic waves with two decades-wide or larger THz spectra) has been explored much more recently than that of narrower spectral regimes (<5 THz) [1,2]. Such an ever-increasing interest has been mainly arousing from the fact that ultrashort THz transients, such as those generated via laser-induced plasma techniques or spintronics emitters [3,4,5,6,7,8,9], feature extremely wide continuous (nearly gap-free) spectra. These pulses are associated to single-cycle electric field waves with time duration of a few hundreds of femtoseconds or less. Such specific properties enable the possibility of expanding the range of applications where THz radiation can be successfully employed, e.g., accurate and non-invasive time-of-flight measurements on ultra-thin (a few micrometers) low-refractive epitaxial films [3,10,11], and decades-wide spectroscopy of many types of materials [12,13,14,15]. Additionally, THz pulses with electric field peaks higher than 100 kV/cm [5,16,17,18] can trigger high-order nonlinear phenomena at THz frequencies [19,20,21]. Among others, this type of strong nonlinear interaction can potentially result in the generation of new frequency components that fall in a spectral region larger than the fundamental THz pulse spectrum [22,23]. Therefore, the implementation of detection techniques with very wide and gap-less THz spectral responses has rapidly become a topic of intense research. Specifically, gas-based detection techniques have been demonstrated to be particularly suitable for operation in the ultra-broadband THz regime. Gases exhibit very low dispersion and do not possess a lattice structure due to the random motion of the composing particles. As such, a gas medium normally shows a rather uniform spectral response across several decades [24]. In a pioneering work, Dai et al. [25] proposed to exploit the so-called THz-field-induced second harmonic (TFISH) generation in gases to implement a field-resolved detection scheme with an almost 10-THz-wide spectral response, named air-*breakdown* coherent detection technique. This technique relies on the nonlinear mixing between the THz and an optical probe beam within an intense plasma spark, generated through tunneling ionization, at the expense of an extremely high optical energy (~500 μJ). A better implementation of this paradigm referred as air-*biased* coherent detection (ABCD) technique, was successively presented by Karpowicz et al. [13]. Here, the authors exploited a heterodyne scheme fed by electrical square wave (AC) biases, to achieve high dynamic ranges and a gap-less spectral response up to 20 THz, without the requirement of generating a plasma channel. Further developments and improvements of the ABCD method dealt with the employment of different type of gases and tailored electrodes, aimed at enhancing the TFISH yield [26,27]. The ABCD technique still represents the benchmark for field-resolved experiments in the ultra-broadband THz regime. However, Li et al. [28] showed that coherent detection of ultra-broadband THz pulses could be also achieved via an all-optical scheme, relying on a homodyne configuration.

Inspired by the mechanism underlying the ABCD technique, we have recently presented a new paradigm named solid-state-biased coherent detection (SSBCD), which is operated through the use of CMOS-process-compatible solid-state devices. As explained in the following section, in the SSBCD device, the nonlinear interaction between the THz and the probe beams is confined into an epitaxial layer of either silica [29] or silicon nitride (SiN) [30], both having a deep-subwavelength thickness (~μm) and biased by a micro-sized metallic slit. The previous operating configuration adopted for the SSBCD techniques relied on a heterodyne scheme, similarly to the ABCD technique, yet allowing for a coherent detection of 10-THz-wide pulses at probe energies and bias voltages four and two orders of magnitude lower than the air-biased method, respectively.

In this work, we show that the SSBCD technique can be also operated via an affordable and simple homodyne scheme. In particular, here we use the same SSBCD devices previously employed in the heterodyne configuration, yet biased with a plain DC (i.e., static) electric field. The advantages demonstrated by the DC-driven homodyne scheme allows for a crucial simplification of the SSBCD scheme, thus making this ultra-broadband THz technique of easier implementation.

## 2. SSBCD Device Working Principle

The SSBCD technique has been extensively discussed in Refs [29,30]. For the sake of completeness, here we recall that the SSBCD method exploits the TFISH mechanism to sense THz radiation [31]. More in details, when a THz and an optical (probe) pulsed beam are both spatially and temporally overlapped, their nonlinear interaction gives rise to a new beam (namely, TFISH beam) closely oscillating at the second harmonic (SH) frequency of the probe beam. The TFISH beam shows an intensity transient (*I_TFISH_*) proportional to the THz field intensity:

(1) $$I_{TFISH}\propto {\left|\chi^{\left(3\right)}I_{P}E_{THz}\right|}^{2}$$

where *χ*^(3)^ is the nonlinear susceptibility of the third-order medium hosting the THz and probe interaction, *I_P_* the probe intensity and *E_THz_* the THz electric field. Since photodetectors are sensitive to *I_TFISH_* rather than the associated electric field (*E_TFISH_*), the TFISH beam has to beat with another beam—oscillating at the probe SH—in order to achieve coherent detection. Since such an additional field (*E_LO_*) is directly generated within the THz-probe interaction region, it is conventionally named “local oscillator (LO)”. In general, such a mixing leads to [28]:

(2) $$I_{SH}^{BEAT}\propto {\left|\chi^{\left(3\right)}I_{P}E_{THz}+E_{LO}\right|}^{2}={\left|\chi^{\left(3\right)}I_{P}E_{THz}\right|}^{2}+2\chi^{\left(3\right)}I_{P}E_{THz}E_{LO}\cos\left(\Delta \phi \right)+{|E_{LO}|}^{2}$$

where Δ*ϕ* = 2*ϕ_P_* − *ϕ_LO_* is the phase difference between the TFISH and LO beams. The second term in Equation (2), resulting from the interaction with the LO beam, is the key factor enabling coherent detection, since it is directly proportional to the THz electric field. The heterodyne and homodyne schemes differ from the strategy adopted to extract such a cross term.

## 3. Heterodyne and Homodyne Schemes

**Figure 1:**
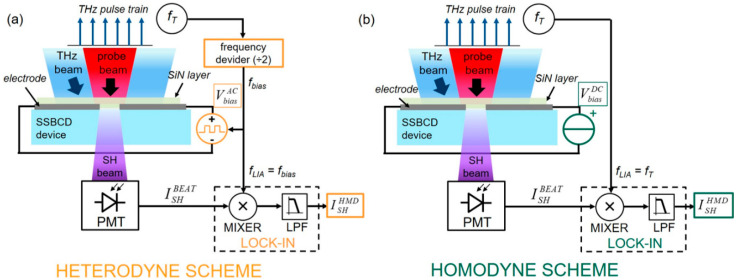
Block diagrams of the solid-state-biased coherent detection (SSBCD) scheme operated via (**a**) a heterodyne and (**b**) a homodyne detection configuration. (**a**) Heterodyne SSBCD: an electronic circuit (orange block) divides by two the THz pulse repetition rate (*f_LO_* = *f_T_*/2) and accordingly generates an AC square wave bias voltage (orange circle), phase-locked to *f_T_*, feeding the SSBCD device. An electrical signal synchronous to *f_LO_* is mixed with the PMT electrical readout $I_{SH}^{BEAT}$ and then is filtered out through a low-pass filter (LPF), so to extract the heterodyne signal *I_HTD_*. Both mixer and LPF are fundamental blocks inside a lock-in amplifier. (**b**) Homodyne SSBCD: a DC bias voltage (green circle) is independently generated by a common power supply. The mixer is fed with a reference signal synchronized to *f_T_*, returning a signal in the base band, filtered out through an LPF, which only acts as a noise attenuation module.

Homodyne (HMD) and Heterodyne (HTD) detection are two terms generally employed to indicate that THz pulse recovery is carried out by exploiting a lock-in-assisted detection configuration, referenced to either the THz pulse repetition rate (*f_T_*) or the LO modulation frequency (*f_LO_*), respectively. Since, the LO beam is generated, in the SSBCD devices, via the external application of a bias electric field (*E_bias_*), i.e., *E_LO_* = *χ*^(3)^*I_P_ E_bias_* [32], for simplicity we will refer to the LO term as *E_bias_*, rather than its intensity. The advantage of this choice will be clear in the next section. In Figure 1, we highlight the differences between the block diagrams for the (a) heterodyne and (b) homodyne configurations. In both schemes, the THz pulsed beam, which is modulated at the chopping frequency *f_T_* is focused into the SSBCD device along with the probe beam. In the heterodyne case—(Figure 1a), an electrical signal synchronous to the THz pulse repetition rate, i.e., with the same frequency *f_T_* and phase *ϕ*, is fed into a modular electronic system, which provides an amplified square wave voltage ($V_{bias}^{AC}$) oscillating at *f_LO_* = *f_T_*/2 and phase-locked to the input signal. Such an AC bias voltage is then applied to the SSBCD device where it generates the required AC-modulated electric field ($E_{bias}^{AC}$). The total SH intensity generated in the SSBCD device is acquired via a photomultiplier tube (generating the electrical signal $I_{SH}^{BEAT}$), and is subsequently sent to a frequency conversion circuit. This module essentially consists in an electronic mixer, where $I_{SH}^{BEAT}$ beats with a reference signal at *f_LO_*, and a low-pass filter. As a result, the first and third contributions in Equation (2) are rejected, thus returning the heterodyne signal *I_HTD_*:

(3) $$I_{HTD}\propto 2{\left(\chi^{\left(3\right)}I_{P}\right)}^{2}E_{THz}E_{bias}^{AC}$$

Equation (3) states that *I_HTD_* is directly proportional to the THz electric field, thus ensuring coherent detection. The frequency conversion circuit (mixer and low-pass filter) is commonly implemented inside a lock-in amplifier (LIA) synchronized to *f_LO_* (see Figure 1a). Besides the well-known noise rejection mechanism inherent to the lock-in acquisition, we highlight that the synchronism between the phase-locked AC bias generator and the LIA is the true key enabling heterodyne detection, which leads to Equation (3). In contrast, in the homodyne scheme (Figure 1b), the SSBCD device is biased with a DC (static) voltage $V_{bias}^{DC}$, giving rise to a DC bias electric field $E_{bias}^{DC}$. Since here the bias is a DC value, neither frequency division nor synchronization with the THz pulse repetition rate is required. Indeed, in the homodyne case, all the contributions to *I_BEAT_* in Equation (2) oscillate at *f_T_* only, and there is no specific term at a different frequency to be filtered out. Therefore, in the homodyne configuration the LIA is directly synchronized to *f_T_* and simply serves as a noise attenuator. Finally, the homodyne readout signal *I_HMD_* is:

(4) $$I_{HMD}\propto {\left[\chi^{\left(3\right)}I_{P}\right]}^{2}\left(E_{THz}^{2}\pm 2E_{THz}E_{bias}^{DC}\right)$$

where the double sign depends on the mutual orientation of the THz and DC bias electric fields. Equation (4) reveals that the homodyne detection is characterized by two main regimes, depending on the THz field strength: for field $E_{bias}^{DC}$ lower or comparable with *E_THz_*, *I_HMD_* shows an intermediate hybrid shape because of the non-negligible contribution stemming from the quadratic incoherent term (∝$E_{THz}^{2}$). However, as $E_{bias}^{DC}$ becomes sufficiently high to satisfy the condition:

(5) $$2E_{bias}^{DC}/E_{THz}>>1$$

the cross term in Equation (4) takes over and detection becomes phase-sensitive.

## 4. Results and Discussion

The integrated devices used to operate the SSBCD technique are uniquely suited to give rise to DC fields sufficiently high to fulfill Equation (5). In particular, this already happens at few tens of volts, thus readily leading to the homodyne coherent detection regime. A detailed description of the SSBCD devices is provided in the Supplementary Materials. We recall that a bias voltage of 100 V applied to the device corresponds to an electric field of $E_{bias}^{DC}$ ≈ 140 kV/cm. As a comparison, breakdown in air usually takes place at ~31 kV/cm. Moreover, the maximum applicable voltage to the SSBCD device before discharge occurs is around 200 V, corresponding to a bias field of ~290 kV/cm. Since the bias voltage can be chosen as a bipolar wave for the heterodyne case, whereas it is unipolar only (with either positive or negative amplitude) for the homodyne scheme, hereinafter we will always refer to peak-to-peak values (V_PP_). This way, we are able to provide a fair comparison between the results achieved in the two configurations. In order to demonstrate the validity of Equations (4) and (5), we placed the SSBCD device at the detection position of an ultra-broadband THz Time-Domain Spectroscopy setup [29], featuring a two-color plasma source fed by a 150 fs, 800 nm, 1 kHz, 1.3 mJ pulsed laser. Such a source emitted ~10-THz-wide single-cycle pulses with a peak amplitude of $E_{THz}^{peak}$ = 170 kV/cm, measured via electro-optic sampling in a 200-µm-thick GaP crystal [33], in a nitrogen-purged atmosphere. The THz electric field strength was adjusted by inserting a pair of wire grid polarizers in the THz beam path, while the probe energy was fixed at 50 nJ for all experiments. It is worth underlying that such a class of SSBCD devices also induces a significant field enhancement (FE) of the impinging THz electric field, previously simulated and experimentally verified [30] to be as high as FE~6. This means that the actual THz electric field in the slit can reach a value significantly higher than that measured in free-space. Therefore, we experimentally estimated the THz field strength for any position of the polarizers by following the strategy detailed in Ref. [30], so to properly compare the values of $E_{bias}^{DC}$ and *E_THz_*.

### 4.1. THz Electric Field Scaling

**Figure 2:**
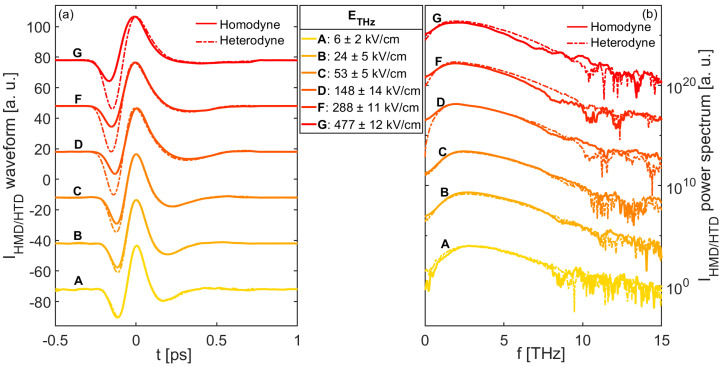
(**a**) Temporal waveforms and (**b**) their corresponding spectra retrieved via the homodyne (solid line) and heterodyne (dashed line) schemes at different THz electric field strengths, and at a fixed bias voltage of $V_{bias}^{DC}$ = 100 V_PP_ ($E_{bias}^{DC}$ ≈ 140 kV/cm). Curves are normalized to their own maxima and vertically shifted for clarity.

The data shown in Figure 2a,b illustrate the temporal waveforms and their corresponding spectra evaluated through a Fast Fourier Transform (FFT) algorithm, respectively. Specifically, such curves were obtained for a fixed $V_{bias}^{DC}$ = 100 V_PP_, while *E_THz_* spanned the range of 6–480 kV/cm (within the slit). Moreover, in both plots, the dashed lines represent the THz waveforms and the spectra reconstructed by using the heterodyne scheme, when operated with a 100 V_PP_ square wave voltage (AC), modulated at 500 Hz. We notice that the homodyne scheme allows for the correct reconstruction of the THz waveforms with a good approximation for *E_THz_* up to ~50 kV/cm. This is consistent with Equation (5), evaluated for $E_{bias}^{DC}$ ≈ 140 kV/cm. The direct comparison between *E_THz_* and as a means to estimate the minimum DC voltage required to achieve coherent detection, demonstrates the potential advantage of our electrically-driven homodyne scheme over optically-biased techniques, where the only directly accessible information is the LO optical power. For higher THz field values, the recorded transient shapes deviate from those acquired with the heterodyne scheme, showing an increasingly pronounced unipolar form (Figure 2a) as a consequence of an incorrect phase recovery. This observation is more evident in the THz spectra shown in Figure 2b, where the overlap between the bandwidths calculated in the homodyne and heterodyne configurations especially worsens at low frequencies, as the THz strength exceeds 50 kV/cm. Moreover, modulations in the spectra appear at high frequencies for very large THz electric fields (>150 kV/cm), while the peaks of the spectra shift towards the origin of the frequency axis. For *E_THz_* > 500 kV/cm (not shown in Figure 2), detection was purely incoherent.

### 4.2. Differential Homodyne Scheme

As previously mentioned, in order to expand the range of field strengths for which THz pulses can be coherently reconstructed, the DC bias voltage should be increased according to Equation (5). However, if *E_THz_* overcomes 100 kV/cm, coherent detection could be achieved (in the current device geometry) by applying a DC voltage exceeding the maximum value that the SSBCD device can safely withstand (i.e., ~200 V_PP_). For example, in Figure S2 of the Supplementary Materials, we show in detail the reshaping effect experienced by the temporal waveforms reconstructed via homodyne SSBCD as a function of the bias voltage. In that case, $V_{bias}^{DC}$ spans the bipolar range between −200 and +200 V_PP_ for a fixed $E_{THz}^{peak}$= 150 kV/cm.

In order to overcome the limitation on the maximum applicable bias voltage and thus attain coherent detection at potentially any THz field strength, we adopted the following strategy. We recall that by performing two different measurements with two bias fields of the same magnitude but with a reversed sign (namely, $E_{bias}^{+DC}=-E_{bias}^{-DC}=\left|E^{DC}\right|$) the two signals are expressed as:

(6) $$I_{HMD}^{+}\propto E_{THz}^{2}+2E_{THz}E_{bias}^{+DC}=E_{THz}^{2}+2E_{THz}\left|E^{DC}\right|$$

(7) $$I_{HMD}^{-}\propto E_{THz}^{2}+2E_{THz}E_{bias}^{-DC}=E_{THz}^{2}-2E_{THz}\left|E^{DC}\right|$$

where the actual sign of the bias electric field is explicitly shown. These two equations, considered separately, describe the measured signal in the *single-ended* homodyne configuration, as investigated throughout the text so far. If we take the difference between Equations (6) and (7), the incoherent terms cancel out, thus leading to an expression which describes the homodyne differential signal $I_{HMD}^{diff}$:

(8) $$I_{HMD}^{diff}=I_{HMD}^{+}-I_{HMD}^{-}\propto 2E_{THz}\left|E^{DC}\right|$$

**Figure 3:**
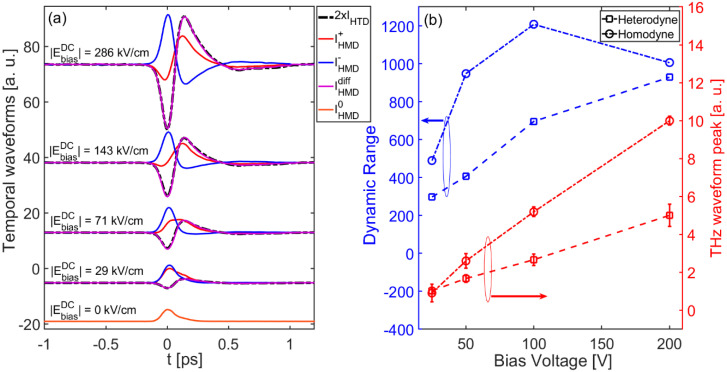
(**a**) Temporal waveforms acquired via the single-ended homodyne configuration, by varying the positive (red dotted curve) and negative (blue dashed line) *E_DC_*, and via the differential scheme (purple solid line), for a fixed *E_THz_* = 150 kV/cm. The dashed black lines represent the THz waveforms recorded via standard heterodyne scheme. Note that the heterodyne curves are multiplied by a factor of 2 for a convenient comparison with those retrieved via the differential homodyne scheme. All the curves are normalized with respect to the peak of the incoherent waveform *I*^0^*_HMD_* (orange solid line) and vertically shifted for clarity. (**b**) Comparison between peak trends (red dots) and DRs (blue dots) corresponding to the case of differential homodyne (squares) and heterodyne (circles) detection. Dashed lines are only used to help the reader to visually link data points.

The differential homodyne signal is always directly proportional to the THz electric field, at any DC bias field value. To demonstrate the validity of Equation (8), in Figure 3a we report the waveforms acquired according to Equations (6) and (7) along with their corresponding difference, obtained for bias voltages spanning the range of |*V^DC^*| = 20–200 V_PP_ (corresponding to the range |*E^DC^*|~29–286 kV/cm) and *E_THz_* = 150 kV/cm. We see that the differential signal features a perfectly reconstructed phase information for each bias voltage value, including those cases where neither $I_{HMD}^{+}$ nor $I_{HMD}^{-}$ transients show a bipolar shape (i.e., for |*E^DC^*| < 71 kV/cm), as proved by the very good overlap with the THz pulse recorded via the heterodyne scheme. The differential homodyne scheme allows us to coherently detect THz pulses with a relatively high peak amplitude, already at bias voltage values as low as 20 V. Figure 3b shows the peak trend and corresponding dynamic range (DR) [34] of the $I_{HMD}^{diff}$ curves as a function of the bias voltage, compared to those measured via the heterodyne SSBCD. Remarkably, the differential scheme provides two additional advantages with respect to the heterodyne configuration. First, the differential signal linearly increases with the bias field, yet showing a slope twice as that found for the heterodyne signal [compare Equations (8) and (2)]. Therefore, the differential scheme allows reconstructing the THz waveform by using an effective bias electric field |*E^DC^|* that is twice the actual value applied to the SSBCD device, thus making detection more sensitive to lower THz field strength. Second, the performance in terms of DR was superior for the homodyne differential scheme, up to a ~2-fold improvement. Indeed, in contrast to the heterodyne case, the noise has to be attributed to laser fluctuations only in the homodyne scheme, since the voltage was generated by a low-noise power supply, providing a very clean DC voltage. Therefore, regardless of the DC voltage polarity, the noise affecting the two measurements follows the same statistics, thus being greatly attenuated in the differential signal (common noise rejection). We observed a drop of the DR at |*V^DC^*| = 200 V_PP_, most likely due to higher order nonlinear effects occurring inside the SSBCD device, when the bias voltage approaches its breakdown threshold, thus resulting in additional noise contributions affecting both $I_{HMD}^{+}$ and $I_{HMD}^{+}$.

### 4.3. Absolute THz Pulse Polarity Recovery

**Figure 4:**
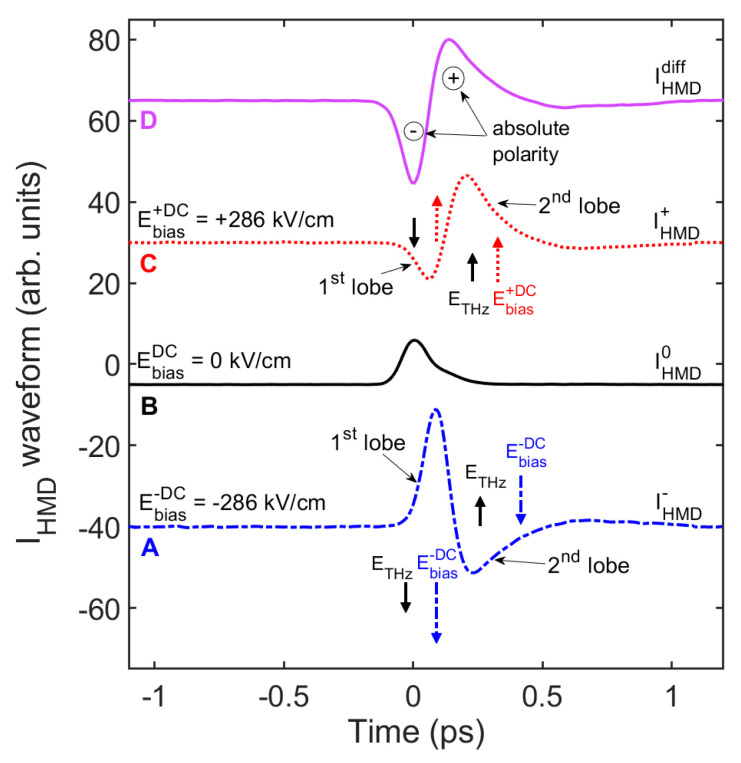
Recovery of the THz pulse absolute polarity. The temporal waveforms retrieved via the SSBCD homodyne scheme shows that, with respect to the non-biased case (curve **B**), the first lobe is amplified by a negative bias (curve **A**), whereas a positive bias causes its attenuation (curve **C**). The opposite occurs for the second lobe. This suggests that the oscillation of the THz electric field wave begins with a “negative” cycle (curve **D**). For all curves, the THz electric field strength is fixed at 150 kV/cm.

In Figure 3, the shape of the recorded waveforms at negative bias voltages is not simply specular with respect to the positive counterparts. In particular, in Figure 4 we focus the attention on the waveforms retrieved for $E_{bias}^{+DC}=-E_{bias}^{-DC}=\left|E^{DC}\right|$ = 286 kV/cm (curves A and C), compared to the purely incoherent and differential waveforms (curves B and D, respectively). Although it is not ultimately possible to detect the exact true form of a THz pulse, due to the non-flat spectral response affecting any type of THz detector, the SSBCD homodyne scheme allows us to obtain a crucial insight regarding the absolute polarity of the THz pulse. In the incoherent curve (B), retrieved for a THz strength of *E_THz_* = 150 kV/cm, the two pulse lobes are merged together and not distinguishable. However, when the 286 kV/cm-bias is applied, the first lobe is amplified by the negative DC field value [$I_{HMD}^{-}\propto E_{THz}^{2}+2(-E_{THz})(-E_{bias}^{DC})>0$, curve A], whereas it is attenuated and even reversed in sign by the positive bias value [$I_{HMD}^{+}\propto E_{THz}^{2}+2(-E_{THz})(+E_{bias}^{DC})<0$, curve C]. The opposite effect occurs for the second lobe. Therefore, considering Equation (4), since the incoherent term must be inherently a positive quantity ($\propto E_{THz}^{2}$, curve B), we conclude that the THz pulse begins to emerge in the presence of a negative lobe, followed by a positive lobe (curve D). Such an information cannot be easily obtained through the heterodyne scheme, since in this case the lock-in amplifier acquires the data to within a ±π-phase uncertainty.

## 5. Conclusions

In conclusion, we demonstrated a particularly convenient implementation of the SSBCD technique, via an affordable homodyne scheme, operated with plain DC bias voltages. This is in contrast to the conventional heterodyne configuration, where sophisticated electronics is necessary to generate amplified, fast-switching AC bias voltages, additionally synchronized to the THz pulse repetition rate. Moreover, the heterodyne scheme relies on the essential filtering action performed by a frequency conversion electronic circuit, such as a lock-in amplifier. Differently, DC bias voltages are easily provided by portable and off-of-the-shelf power supplies, generating clean voltages with extremely low noise fluctuations, while no frequency conversion is required to reach the coherent detection regime. Furthermore, the differential homodyne scheme enables a pure THz coherent detection at significantly low DC bias voltage values with higher dynamic ranges (by a factor of 2), implying an improved sensitivity especially at low THz strengths, with respect to the heterodyne counterpart. Additionally, the absolute polarity of the THz pulse can be retrieved by simply reversing the sign of the DC bias voltage. This feature may be exploited in applications based on the monitoring of the carrier-envelope phase of single-cycle THz pulses [35,36]. The DC biased homodyne scheme enables the performing of the SSBCD technique with very simple and cost-effective equipment, thus allowing non-specialized users (e.g., biologists, chemists, material scientists) to benefit in their THz field-resolved applications from an ultra-broadband coherent detection method. Finally, we envision that our technique will be particularly suitable to detect THz pulses generated via various techniques exploiting the emerging technology of high-repetition rate (>100 kHz) Ytterbium lasers [37,38,39,40].

## 6. Patents

“Method and a system for homodyne solid-state biased coherent detection of ultra-broadband terahertz pulses” No. US17/086,814 (2020).

## Data Availability

The data presented in this study are available upon a reasonable request from the corresponding authors.

## Supplementary Materials

The following are available online at https://www.mdpi.com/2079-4991/11/2/283/s1, Figure S1: Layout of the SSBCD device, Figure S2: DC electric field scaling.
