# Supplementary material for: Homodyne Solid-State Biased Coherent Detection of Ultra-Broadband Terahertz Pulses with Static Electric Fields
Source: Nanomaterials (Basel). 2021 Jan 22;11(2):283. doi: 10.3390/nano11020283 (PMC7911412; doi:10.3390/nano11020283)
Supplement: Supplementary file 1 [file nanomaterials-11-00283-s001.pdf]

Supplementary Materials

Homodyne Solid-State-Biased Coherent Detection of Ultra-Broadband Terahertz Pulses with Static Electric Fields

1. Design and fabrication of the SSBCD devices

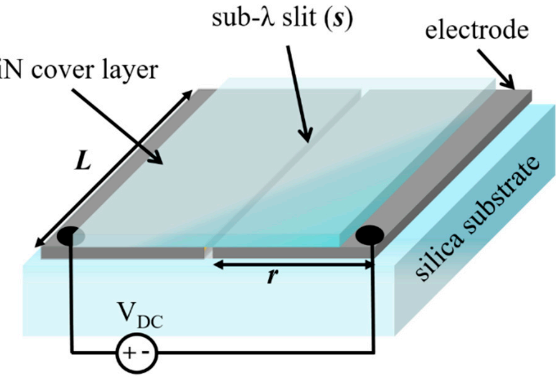

Figure S1. Layout of the SSBCD device.

The SSBCD devices employed in this work have been fabricated via standard CMOS technology. Their geometrical structure is presented in Figure S1. The fabrication process is relatively simple and starts with the sputtering deposition on a 0.5-mm-thick fused silica substrate of a stack of three metallic layers. These are constituted of a 100-nm-thick slab of aluminum (Al) sandwiched between two 30-nm-thick layers of chromium (Cr). The Cr layers are necessary to ensure the proper adhesion between the metallic layers and the substrate underneath, as well as with the subsequently deposited dielectric material. In order to realize the two electrodes, the whole metallic pad is first processed via direct-write laser lithography and then defined via wet etching techniques, so to ultimately form a 1.0±0.1 μm-wide slit (denoted as *s* in Figure S1). Finally, a 1-μm-thick silicon nitride (precisely, *plasma nitride* SiN [1]) cover layer is deposited via plasma-enhanced chemical vapor deposition (PECVD) over the whole structure and then patterned by direct-write laser lithography and plasma etching to clear the external region of the electrodes, in order to bias the slit via a pair of contact wires welded on them. The cover layer thickness was chosen in such a way to prevent the occurrence of discharges induced by the high bias electric fields, which spreads out of the slit towards the air above the covering material. In particular, with the aid of numerical simulations [2,3], we found out that, for the structure in Figure S1, a minimum thickness comparable in value with the slit width guarantees safe operating conditions up to a bias voltage of 200 V_{PP}. The aluminum pad sizes were chosen as $r = L = 2$ mm, so to completely gather the focused THz beam (the waist size of which is usually much smaller than 1 mm for a 10-THz-wide THz pulse spectrum).

We would like to stress the fact that even though silicon nitride as a stoichiometric film (Si_3N_4) commonly shows some phonon resonances around and above 15 THz [4], in our case this circumstance does not represent an issue affecting the detection of ultra-broadband THz pulses, because of the following reasons:

- **The SSBCD devices have been optimized for operation in the 0.1–10 THz frequency region**, a two-decade wide spectral window often referred as the "THz range". Indeed, we have tested the SSBCD devices with pulses featuring a 10-THz-wide spectrum as emitted by a two-color plasma source fed by a ~150 fs pulsed laser. This type of pulses does not appreciably contain frequency components falling in the far-infrared range (>12 THz), thus potential material resonances cannot play any role at these THz wavelengths.

- **The SiN film deposited on the SSBCD has a *deep sub-wavelength* thickness of only 1 μm.** Due to such a dramatically reduced thickness, the THz pulse propagates through the SiN film for an extremely short length, comparable to a fraction of the central wavelength of the THz pulse (~300 μm). At this scale, the terahertz-field-induced second harmonic generation process occurring within the slit is not expected to be affected by any constraint related to either phase-matching or lattice resonances.

- **The silicon nitride film is grown via PECVD at a low temperature of 300 °C**. PECVD films deposition at low temperature does not lead to stoichiometric films, rather the resulting silicon nitride is particularly hydrogen-rich and exhibits a totally amorphous structure [1]. Moreover, the SiN layer is directly deposited mostly over a metallic thin film. Differently from a silicon substrate, the lattice structure of a metal is not an optimal seed for the SiN epitaxial growth. Indeed, the metal lattice constant is completely mismatched with respect to silicon nitride, thus further preventing the formation of an ordered crystalline structure, while inducing the accumulation of a certain amount of stress in the atom bonds. These growing conditions imply a strong relaxation of the phonon resonance strengths [5], provided that such resonances still takes place within the amorphous film.

In light of these observations, we can safely affirm that the highly amorphous and thin SiN films used in this class of SSBCD device makes them particularly suitable to perform gap-free detection of ultra-broadband THz pulses.

2. Bias voltage scaling

In a similar manner to the study detailed in the main manuscript, section II, here Figure S2a,b, show the temporal waveforms and their calculated spectra, respectively, obtained at a fixed THz field strength of E_{THz} = 150 kV/cm in the slit and a bias electric field spanning the bipolar range between -283 and +283 kV/cm (-200 and +200 V_{PP} in terms of bias voltage). For positive biases, we clearly see that the unipolar curve achieved for $E_{DC} \equiv 0$ (which resembles the envelope of the THz pulse intensity), gradually turns into a bipolar transient as the bias DC field increases. Accordingly, the bell-shaped spectrum reconstructed without any applied bias shows a maximum on the origin of the frequency axis, since its corresponding transient has a non-null mean value. Consistent with the corresponding temporal waveforms, the peak of the spectrum moves towards higher frequencies as soon as E_{DC} overcomes E_{THz}, while the whole spectrum shape tends to resemble the curve retrieved in the heterodyne case. As mentioned in the main manuscript, the application of a bias voltage with opposed polarity generates temporal transients with non-specular shapes, due to the reciprocal orientation of THz and bias electric fields (see section VI in the main text).

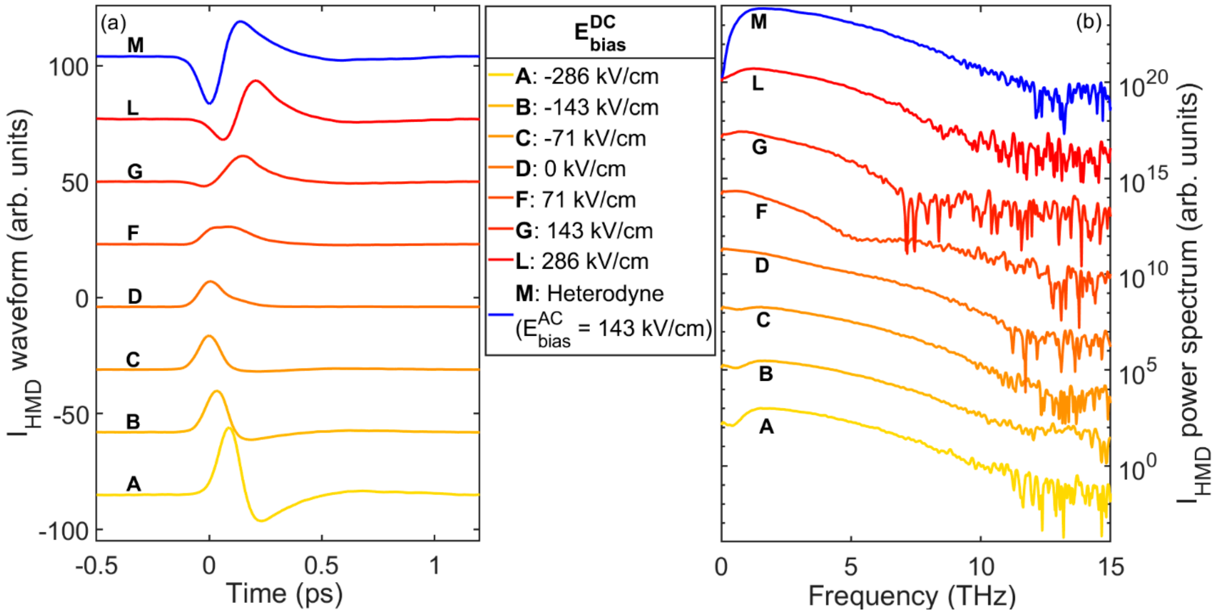

Figure S2. DC electric field scaling. (**a**) Temporal waveforms and (**b**) their corresponding spectra recorded via the homodyne configuration at different DC bias electric field values spanning the bipolar range between -283 and +283 kV/cm and for a fixed THz electric field strength of 150 kV/cm. The highest curve in each plot (indicated as M, blue solid line) represents (**a**) the THz pulse and (**b**) the spectrum acquired via heterodyne detection, shown for comparison. The curves are normalized with respect to their own maximum and vertically shifted for clarity.

References

1. May, G.S.; Sze, S.M. *Fundamentals of semiconductor fabrication*; Wiley, 2004; ISBN

9780471232797.

2. Tomasino, A.; Mazhorova, A.; Clerici, M.; Peccianti, M.; Ho, S.-P.; Jestin, Y.; Pasquazi, A.; Markov, A.; Jin, X.; Piccoli, R.; et al. Solid-state-biased coherent detection of ultra-broadband terahertz pulses. *Optica* **2017**, *4*, 1358, doi:10.1364/OPTICA.4.001358.

3. Tomasino, A.; Piccoli, R.; Jestin, Y.; Delprat, S.; Chaker, M.; Peccianti, M.; Clerici, M.; Busacca, A.; Razzari, L.; Morandotti, R. Invited Article: Ultra-broadband terahertz coherent detection via a silicon nitride-based deep sub-wavelength metallic slit. *APL Photonics* **2018**, *3*, 110805, doi:10.1063/1.5052628.

4. Kischkat, J.; Peters, S.; Gruska, B.; Semtsiv, M.; Chashnikova, M.; Klinkmüller, M.; Fedosenko, O.; Machulik, S.; Aleksandrova, A.; Monastyrskyi, G.; et al. Mid-infrared optical properties of thin films of aluminum oxide, titanium dioxide, silicon dioxide, aluminum nitride, and silicon nitride. *Appl. Opt.* **2012**, *51*, 6789, doi:10.1364/AO.51.006789.

5. Bass, M.; Li, G.; Van Stryland, E.W. *Handbook of optics. Volume IV, Optical properties of materials, nonlinear optics, quantum optics*; McGraw-Hill, 2010; ISBN 0071498923.
